# Effects of Ni precursors on the formation of Mg–Fe–Ni intermetallic hydrides, kinetics, and reversibility

**DOI:** 10.1039/d3ra01914d

**Published:** 2023-06-05

**Authors:** Palmarin Dansirima, Sophida Thiangviriya, Praphatsorn Plerdsranoy, Narong Chanlek, Rapee Utke

**Affiliations:** a School of Chemistry, Institute of Science, Suranaree University of Technology Nakhon Ratchasima 30000 Thailand rapee.g@sut.ac.th; b Synchrotron Light Research Institute (Public Organization) Nakhon Ratchasima 30000 Thailand

## Abstract

This work focuses on the effects of Ni precursors (metallic Ni or Mg_2_NiH_4_) on the formation of Mg–Fe–Ni intermetallic hydrides as well as their de/rehydrogenation kinetics and reversibility. After ball milling and sintering, the formation of Mg_2_FeH_6_ and Mg_2_NiH_4_ are found in both samples, while MgH_2_ is observed only in the sample with metallic Ni. Both samples show comparable hydrogen capacities of 3.2–3.3 wt% H_2_ during the 1^st^ dehydrogenation, but the sample with metallic Ni decomposes at a lower temperature (Δ*T* = 12 °C) and shows faster kinetics. Although phase compositions after dehydrogenation of both samples are comparable, their rehydrogenation mechanisms are different. This affects the kinetic properties upon cycling and reversibility. Reversible capacities of the samples with metallic Ni and Mg_2_NiH_4_ during the 2^nd^ dehydrogenation are 3.2 and 2.8 wt% H_2_, respectively, while those during the 3^rd^–7^th^ cycles reduce to ∼2.8 and 2.6 wt% H_2_, respectively. Chemical and microstructural characterizations are carried out to explain de/rehydrogenation pathways.

## Abstract 2

Different Ni precursors (metallic Ni or Mg_2_NiH_4_) used for synthesizing Mg–Fe–Ni based hydrides affect micro-scaled distribution and contacts of the reactive phases upon cycling, altering H_2_ ab/desorption mechanism and reversible capacities.

## Graphical abstract

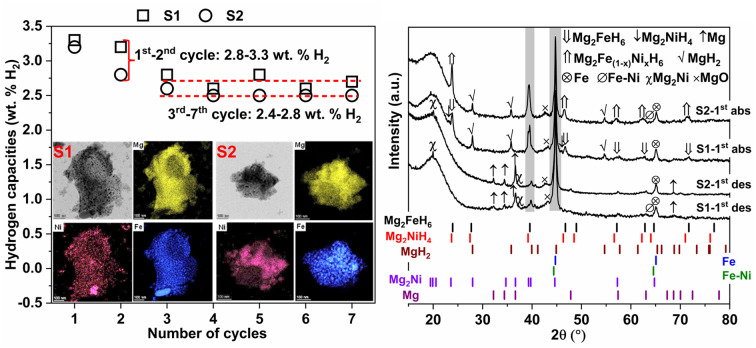

## 1. Introduction

The intermetallic hydride of Mg_2_FeH_6_ has been considered for hydrogen storage applications due to the highest volumetric hydrogen density (150 kg m^−3^) and relatively high gravimetric hydrogen density (5.5 wt% H_2_).^1,2^ Also, its high reaction enthalpy (∼90 kJ per mol H_2_) as well as high volumetric and gravimetric energy densities (0.49 kW h L^−1^ and 0.55 kW h kg^−1^, respectively) are suitable for the thermochemical energy storage medium.^1,3–6^ However, Mg_2_Fe intermetallic alloy is thermodynamically unfavorable and the significant difference in density and melting points of Mg and Fe hinders the formation of homogeneous alloys *via* metallurgical methods.^7,8^ Several procedures, such as thermal processes, mechanical milling, cold rolling, and high-pressure compressions have been applied to Mg + Fe or MgH_2_ + Fe mixtures for Mg_2_FeH_6_ syntheses.^9–15^ Hydrogenation of Mg to MgH_2_ catalyzed by Fe was first found at ∼200 °C and the obtained MgH_2_ further reacted with Fe to form Mg_2_FeH_6_ only at high temperature (∼350 °C) due to kinetic restriction from solid-solution diffusion processes.^16,17^ Moreover, reversibility of Mg_2_FeH_6_*via* the reaction between MgH_2_ and Fe required high operating temperatures (*T* = 375–445 °C) to achieve reasonable hydrogen capacity.^9,15^

Quaternary intermetallic hydrides *via* partial substitution of transition metals (TMs) for Fe in Mg_2_FeH_6_ to form Mg_2_Fe_(1−*x*)_TM_*x*_H_6_ (TM = Cr, Ni, Mn, Co, and Y) have been proposed to enhance kinetics and reversibility. The samples were prepared by (i) milling MgH_2_ with the plain steel containing TM impurities (*e.g.*, 316L stainless steel and γ-Fe(Ni) nanoparticles)^8,18,19^ and (ii) compositing TMs in metallic form or compounds with Mg + Fe, MgH_2_ + Fe, or Mg_2_FeH_6_.^20–25^ These processes increased Mg_2_FeH_6_ yield with the improved kinetic properties and reversibility. Immediate reaction between MgH_2_ and 316L SS *via* either reactive ball milling under hydrogen pressure or ball milling under Ar atmosphere and annealing under hydrogen pressure resulted in partial substitution of Fe with Cr and Ni to form Mg_2_(Fe, Cr, Ni)H_*x*_.^8,18^ Such a faster reactivity with respect to pure iron was induced by martensitic transformation during ball milling and the presence of Ni in the system. Moreover, Mg_2_Fe(Ni)H_6_ with tangled nanowire morphology prepared using coarse-grained Mg powder and γ-Fe(Ni) nanoparticles showed lower desorption temperature by 20 °C as compared with Mg_2_FeH_6_.^19^ Catalytic effects on hydrogenation of Ni and Fe as well as comparable fcc lattice of γ-Fe(Ni) and Mg_2_FeH_6_, shortening Fe diffusion distance favored the formation of Mg_2_Fe(Ni)H_6_. Besides, NiFe-based catalysts favored hydrogen adsorption kinetics, resulting in the enhanced hydrogen evolution capability.^26,27^ Transition metal complex deuterides of Mg_2_Fe_*x*_Co_(1−*x*)_D_*y*_ (*x* = 0–1 and *y* = 5–6) prepared by reactive ball milling revealed comparable deuterium desorption temperatures at all compositions, but reversible reaction (*T* = 400 °C under 30 bar H_2_) with the enhanced kinetics was detected from Mg_2_Fe_0.5_Co_0.5_H_5.5_.^21^ Theoretical studies reported destabilization of Mg_2_FeH_6_, *i.e.*, reduction of formation energy and desorption temperature *via* substitution of Fe with Ni, Co, and Mn.^20^ The most significant reduction of desorption enthalpy was expected from Mg_2_Fe_0.75_Ni_0.25_H_6_ (27.7 kJ per mol H).

Among Mg–Fe–TM intermetallic hydrides, Mg–Fe–Ni–H system shows remarkable hydrogen sorption kinetics, meanwhile all metallic compositions (Mg, Fe, and Ni) are inexpensive. From our previous work, Mg_2_Fe_0.75_Ni_0.25_H_6_ formed during dehydrogenation of 20 wt% Ni-doped Mg_2_FeH_6_ showed excellent reversible hydrogen capacities with respect to as-prepared Mg_2_FeH_6_, for example, hydrogen reproduction during the 2^nd^ cycle increased from 78 to 85%.^23^ Besides, Ni-substituted contents in Mg_2_FeH_6_ was optimized by varying Mg_2_FeH_6_ : Mg_2_NiH_4_ mole ratios to obtain Mg_2_Fe_(1−*x*)_Ni_*x*_H_6_ with the best kinetics.^25^ It was found that dehydrogenation kinetics and reversibility were enhanced with Ni-substituted contents, and the most stable composition upon cycling was *x* ∼ 0.5 (Mg_2_Fe_0.5_Ni_0_._5_H_6_). From these reports, it was found that different starting materials could alter Ni substitution degree in Mg_2_FeH_6_, *i.e.*, 25 and 26–47% for the samples prepared from metallic Ni + MgH_2_ and Mg_2_FeH_6_ + Mg_2_NiH_4_, respectively. In this work, we would like to extend our study on the effects of Ni precursors on the formation and reversibility of Mg_2_Fe_(1−*x*)_Ni_*x*_H_6_. Two sample sets with the same stoichiometry of *x* = 0.25 using MgH_2_ + Fe + Ni and MgH_2_ + Fe + Mg_2_NiH_4_ mixtures as starting materials are ball milled and sintered under hydrogen pressure. De/rehydrogenation kinetics, reversibility, and hydrogen exchange pathways are investigated. Microstructural analyses are carried out to explain the effects of distribution and contacts among the reactive phases in nanometer range on hydrogen sorption mechanism.

## 2. Experimental

### 2.1 Sample preparation

Mg powder (≥99.0%, Sigma-Aldrich) was hydrogenated at 350 °C under 38–40 bar H_2_ for 12 h and milled for 1 h 30 min using a Retsch™ PM 100 Model Planetary Ball Mills. The rotational speed and the ball-to-powder weight ratio (BPR) were 500 rpm and 10 : 1, respectively. Hydrogenation and ball milling under similar conditions were repeatedly carried out until hydrogenation was complete to obtain as-prepared MgH_2_. Ni powder (99%, Alfa Aesar) was milled with as-prepared MgH_2_ under 1 : 2 mole ratio using milling time, BPR, and rotational speed of 5 h, 10 : 1, and 500 rpm, respectively. Hydrogenation of 2MgH_2_–Ni mixture was done at 350 °C under 40 bar H_2_ for 12 h to obtain as-prepared Mg_2_NiH_4_. As-prepared MgH_2_ was milled with the powder samples of Ni and Fe (99.9%, Sigma-Aldrich) with the mole ratio of 8 : 3 : 1 (MgH_2_ : Fe : Ni) for 7 h 30 min using BPR and rotational speed of 15 : 1 and 500 rpm, respectively. The obtained mixture was sintered at 400 °C under 38–40 bar H_2_ for 48 h to obtain MgH_2_–Fe–Ni composite, denoted as S1. Fe powder was milled with as-prepared samples of MgH_2_ and Mg_2_NiH_4_ with the mole ratio of 6 : 3 : 1 (MgH_2_ : Fe : Mg_2_NiH_4_) and the mixture was sintered under similar condition with S1 to produce MgH_2_–Fe–Mg_2_NiH_4_ composite, denoted as S2. The powder samples of S1 and S2 were heated to 500 °C and rehydrogenated at 350 °C under 40 bar H_2_ for 12 h to obtain S1′ and S2′, respectively.

### 2.2 Characterizations

Phase compositions of as-prepared and de/rehydrogenated samples were characterized by powder X-ray diffraction (PXRD) at ambient temperature using a Bruker D8 ADVANCE with Cu K_α_ radiation (*λ* = 1.5406 Å), a current of 40 mA, and a voltage of 40 kV. The powder sample was packed in an airtight sample holder covered with a poly(methyl methacrylate) dome in a nitrogen-filled glove box. The diffractogram was collected in the 2*θ* range, scanning step, and acquisition time of 10–80°, 0.02° s^−1^, and 400 s per step, respectively. Dehydrogenation profiles were investigated by differential scanning calorimetry (DSC) and thermogravimetry (TG) using a Netzsch STA449F3 Jupiter. The powder sample of 20–30 mg was heated from room temperature to 500 °C (5 °C min^−1^) under N_2_ flow (50 mL min^−1^). The relative signal of H_2_ released from the sample was characterized by mass spectroscopy (MS) using a Netzsch QMS 403C.

X-ray photo-electron spectroscopy (XPS) experiments were carried out at the SUTNANOTEC-SLRI Joint Research Facility, Synchrotron Light Research Institute (Public Organization), Thailand. A PHI5000 Versa Probe II (ULVAC-PHI Inc., Japan) with Al Kα (1.486 keV) radiation as an excitation source was used for characterizations. The powder samples were deposited on the sample holder using carbon glue tape in the glove box. Prior to the measurements, the samples were placed in the high vacuum chamber (1 × 10^−8^ mbar) for 2 h. The high-resolution scan of each element was collected using a pass energy of 46.95 eV and a step size of 0.05 eV. Dual-beam charge neutralization (low energy electron and ion beam) method was used to minimize sample charging. The binding energy was calibrated with respect to the C 1s peak (284.8 eV). The data was processed and analyzed by using a MultiPak software version 9.6.0 (ULVAC-PHI, Japan). Peak fitting was performed after Shirley background subtraction. Symmetrical Gaussian–Lorentzian function was used to approximate the line shapes of the fitting components.

De/rehydrogenation kinetics and reversibility were studied using a test station automatically controlled by the program developed in a Labview® environment.^28,29^ Two K-type thermocouples (TCs, −250–1300 °C, SL heater) were used to control and measure the system and sample temperatures during the experiments. Hydrogen release and supply during de/rehydrogenation were controlled by the direct-acting plunger solenoid valves (Type 0255, Bürkert) and the system pressure was detected by a pressure transducer with an operating range of 0–3000 psig (an OMEGA Engineering PX309-3KGI). Hydrogen content desorbed was measured using a mass flow controller (MFC, 0–0.1 standard L min^−1^ (SLM), a Bronkhorst EL-FLOW selected F-201CV). The signals of temperature, pressure, and mass flow rate were transferred to the computer using the module data loggers (a NI USB-6009, National Instruments and an AI210, Wisco). Hydrogenation was done under isothermal condition at the setting temperature (*T*_set_) of 315 °C under 10–16 bar H_2_, while dehydrogenation was carried out at *T*_set_ = 315 °C by releasing hydrogen through MFC with the flow rate of 0.09 SLM. The volume of hydrogen desorbed was obtained from integrating the peak area of hydrogen flow rate (SLM) *versus* time (min) plots. The hydrogen storage capacity was calculated by the following equations.1
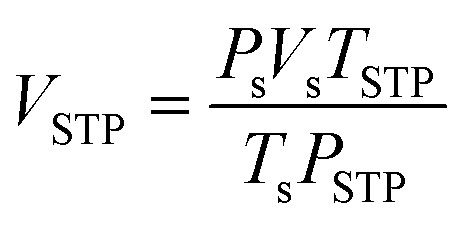
2
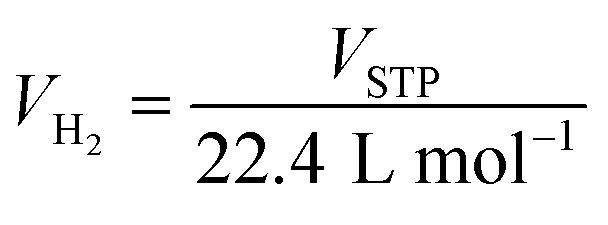
3
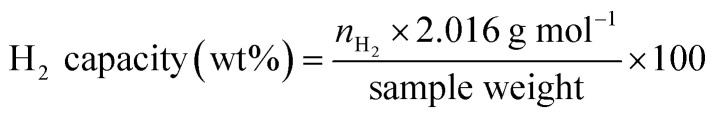
where *V*_STP_ (L) and *V*_s_ (SLM) are the volumes of hydrogen gas at the standard temperature and pressure condition (STP, *T*_STP_ = 273.15 K and *P*_STP_ = 1.0133 bar) and at the standard condition of MFC (*T*_s_ = 296.15 K and *P*_s_ = 1.0156 bar), respectively. *n*_H_2__ (mol) is hydrogen moles and standard molar volume is 22.4 L mol^−1^.

Morphology and microstructure were characterized by transmission electron microscopy (TEM) technique using a Thermo Scientific TALOS F200X coupled with an energy dispersive X-ray spectroscopy (EDS) micro-analysis. An accelerating voltage of 200 kV was used. Sample preparation was done by ultrasonic dispersion of the powder sample in ethyl alcohol (99% AR grade, RCI Labscan) for 10–15 min and dropping onto a carbon grid.

## 3. Results and discussion

**Fig. 1:**
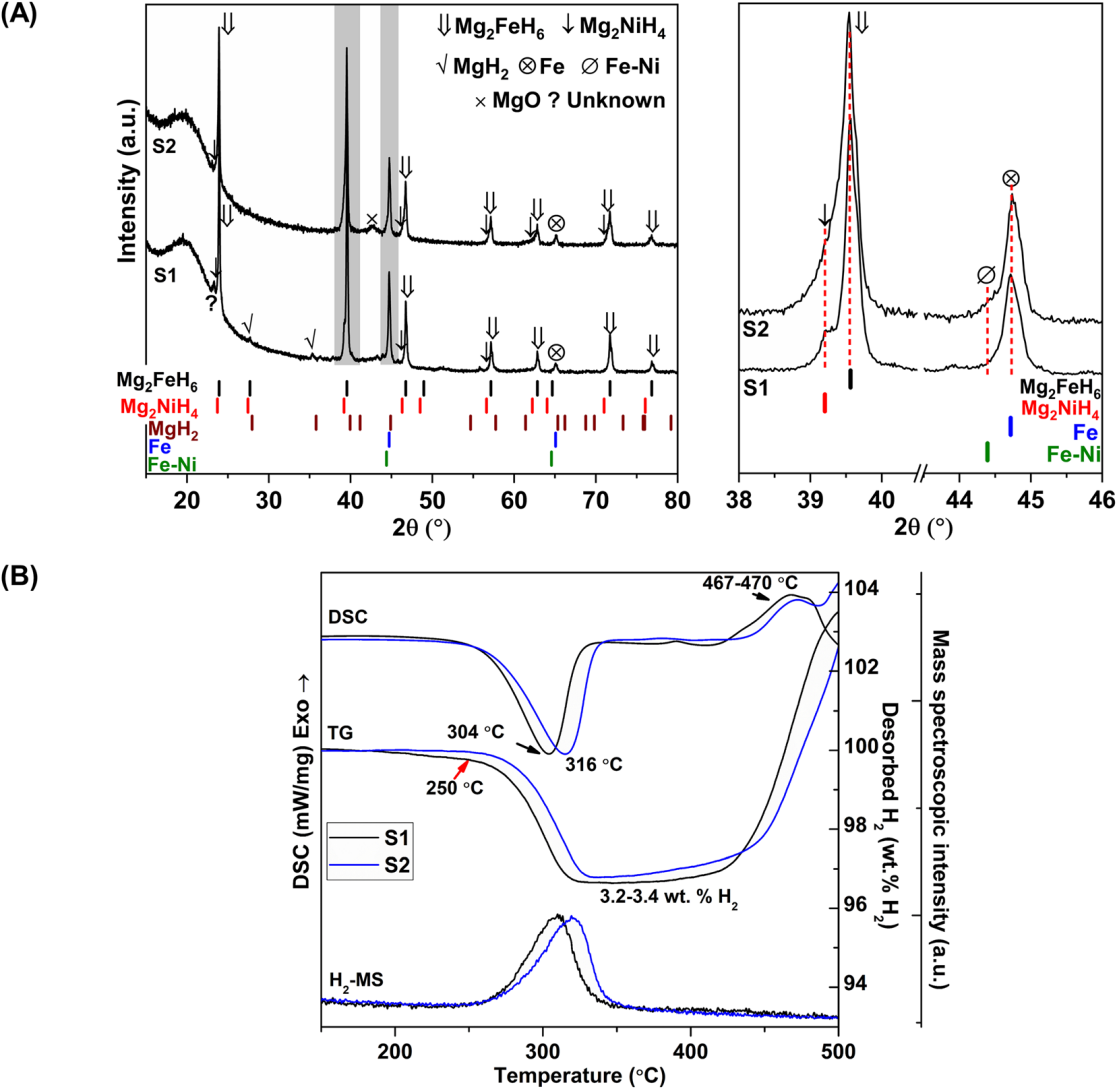
PXRD spectra (A) and simultaneous DSC-TG-MS results (B) of as-prepared S1 and S2.

Chemical compositions of as-prepared S1 and S2 are characterized by PXRD technique. From Fig. 1A, PXRD spectra of S1 and S2 show the diffractions of Mg_2_FeH_6_, Mg_2_NiH_4_, Fe, and MgO as well as MgH_2_ and Fe–Ni alloy^30^ from S1 and S2, respectively. Upon milling and sintering, the formations of Mg_2_FeH_6_ and Mg_2_NiH_4_ confirm hydrogenation of MgH_2_ + Fe (eqn (4)) and Mg_2_Ni (eqn (5)), while that of Fe–Ni alloy is solid solution of Fe and Ni.^30^ MgO is obtained from oxidation of Mg-containing phases with oxygen and/or humidity.

4 2MgH_2(s)_ + Fe_(s)_ + H_2(g)_ → Mg_2_FeH_6(s)_

5 Mg_2_Ni_(s)_ + 2H_2(g)_ → Mg_2_NiH_4(s)_

Dehydrogenation of S1 and S2 is investigated by simultaneous DSC-TG-MS experiments. From Fig. 1B, as-prepared S1 and S2 show single-step decomposition at comparable onset dehydrogenation temperatures of ∼250 °C. The main desorption temperatures of S1 and S2 are 304 and 316 °C, respectively. Hydrogen storage capacities of both samples are comparable in the range of 3.2–3.4 wt% H_2_ (Fig. 1B). Deficient hydrogen capacities with respect to pristine Mg_2_FeH_6_ (5.40 wt% H_2_)^23^ and Mg_2_NiH_4_ (3.4–3.6 wt% H_2_)^31^ are described by the formation of unreacted Fe and Fe–Ni alloy in as-prepared samples (Fig. 1A).

**Fig. 2:**
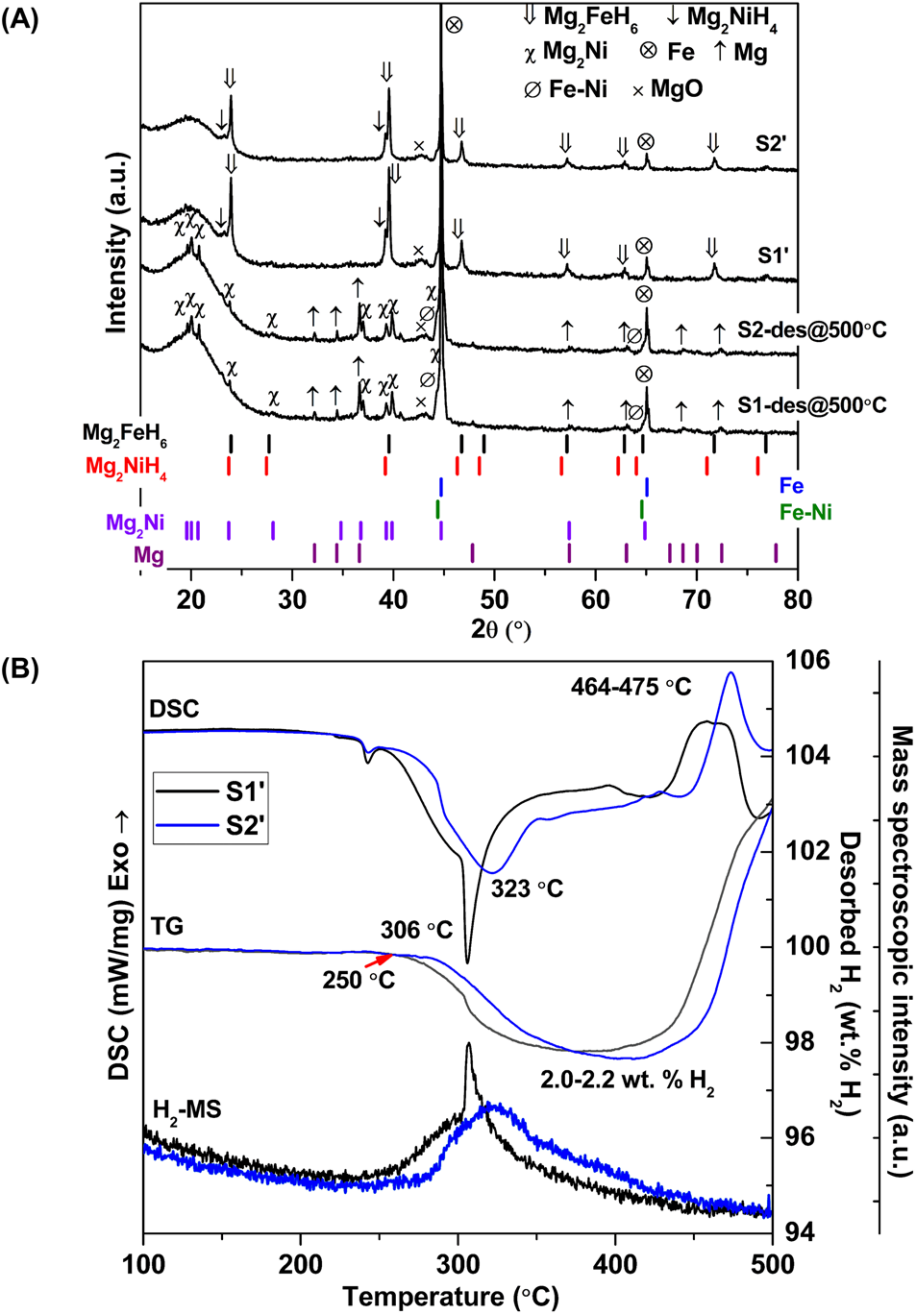
PXRD spectra (A) and simultaneous DSC-TG-MS results (B) after dehydrogenation at 500 °C of S1 and S2 as well as S1′ and S2′.

Considering DSC and TG profiles of S1 and S2, the exothermic event and the weight-gain signals after 450 °C are observed (Fig. 1B). Chemical compositions of S1 and S2 after dehydrogenation at 500 °C and rehydrogenation (S1′ and S2′) are investigated by PXRD technique. From Fig. 2A, PXRD spectra of desorbed S1 and S2 (*T* = 500 °C) show comparable diffractions of Mg_2_Ni, Mg, Fe–Ni alloy, and Fe. Thus, the exothermic peaks at *T* > 450 °C (Fig. 1B) belong to the formation of Mg_2_Ni and Fe–Ni alloy. For S1′ and S2′, similar diffractions of Mg_2_FeH_6_, Mg_2_NiH_4_, and unreacted Fe are observed (Fig. 2A). Dehydrogenation of S1′ and S2′ is characterized by simultaneous DSC-TG-MS experiments. From Fig. 2B, S1′ and S2′ reveal comparable onset and main dehydrogenation temperatures (250 and 306–323 °C, respectively) to those of S1 and S2 (250 and 304–316 °C, respectively) (Fig. 1B). However, storage capacities of S1′ and S2′ (2.0–2.2 wt% H_2_) are significantly lower than those of S1 and S2 (3.4–3.4 wt% H_2_). This is because significant amount of unreacted Fe after dehydrogenation at 500 °C is irreversible after rehydrogenation into S1′ and S2′ (Fig. 2A).

**Fig. 3:**
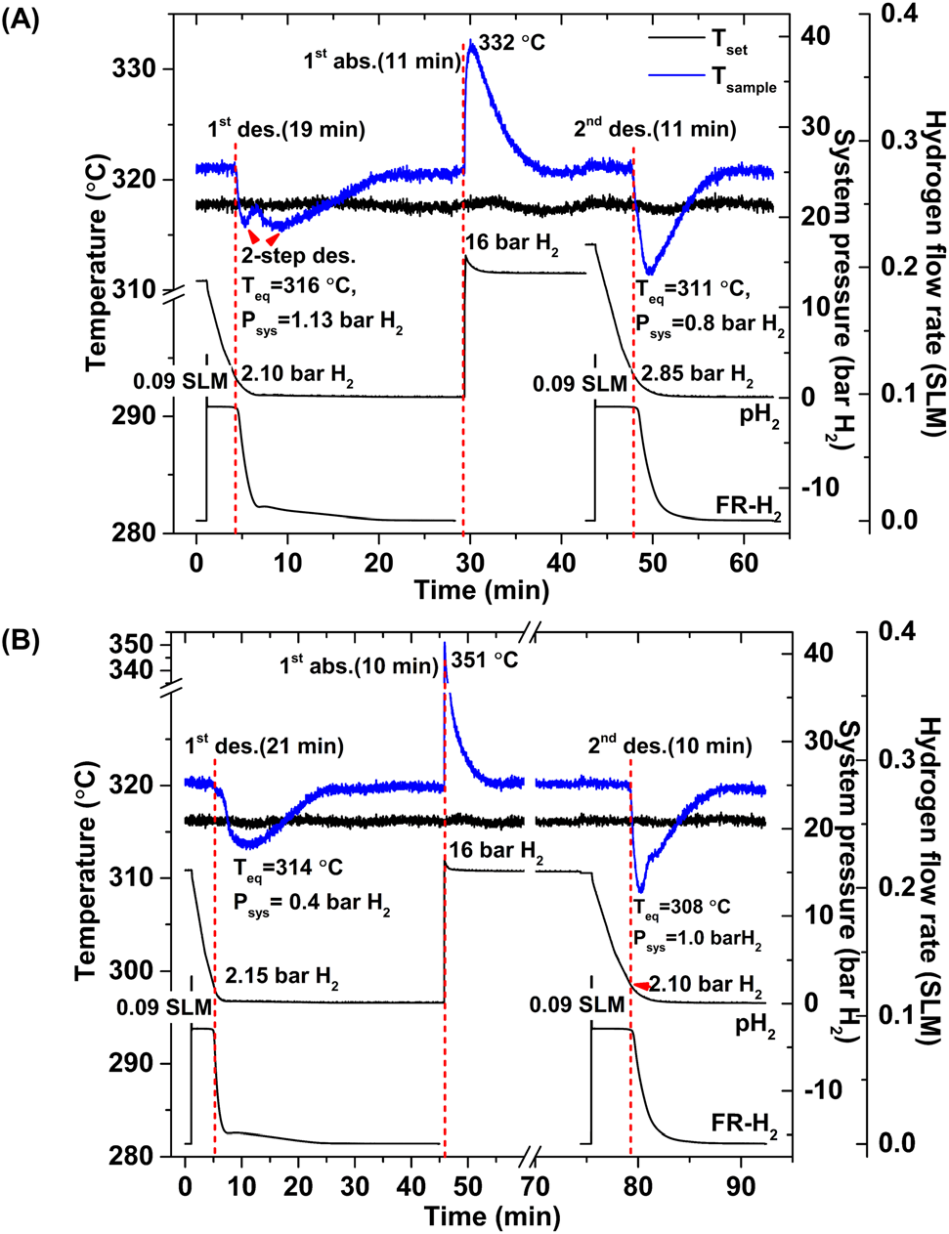
Temperature, pressure, and mass flow rate profiles during de/rehydrogenation of S1 (A) and S2 (B).

According to greater hydrogen capacities and lower dehydrogenation temperatures, further studies focus on dehydrogenation performance, reversibility, and reaction pathways of S1 and S2. Hydrogen absorption and desorption are carried out at isothermal condition (*T*_set_ = 315 °C) under the system pressure (*P*_sys_) of 0–16 bar H_2_. Prior to the measurements, as-prepared samples of S1 and S2 are heated from room temperature to 315 °C under 15 bar H_2_ to prevent dehydrogenation. Once reaching isothermal condition, dehydrogenation begins with releasing hydrogen through MFC using the constant mass flow rate of 0.09 SLM (Fig. 3). During 0–10 min, the 1^st^ endothermic dehydrogenation of S1 and S2 starts at the system pressure (*P*_sys_) of ∼2 bar H_2_, confirmed by the reduction of sample temperature (*T*_sample_) (Fig. 3). Complete dehydrogenation of both samples is obtained within 19–21 min, shown as the elevated *T*_sample_ to the initial temperature. From Fig. 3A, S1 reveals rapid temperature reduction to equilibrium temperature (*T*_eq_) of 316 °C under *P*_sys_ = 1.13 bar H_2_ with two-step decomposition, possibly belonging to MgH_2_, Mg_2_FeH_6_ and Mg_2_NiH_4_. For S2, slow temperature reduction to *T*_eq_ = 314 °C under *P*_sys_ = 0.4 bar H_2_ is found with the single-step dehydrogenation of the mixed Mg_2_NiH_4_ + Mg_2_FeH_6_ (Fig. 3B). At *T*_eq_ = 314–316 °C, the equilibrium pressures (*P*_eq_) of Mg_2_FeH_6_ and Mg_2_NiH_4_ are ∼1.5 and 4 bar H_2_, respectively.^32^

**Fig. 4:**
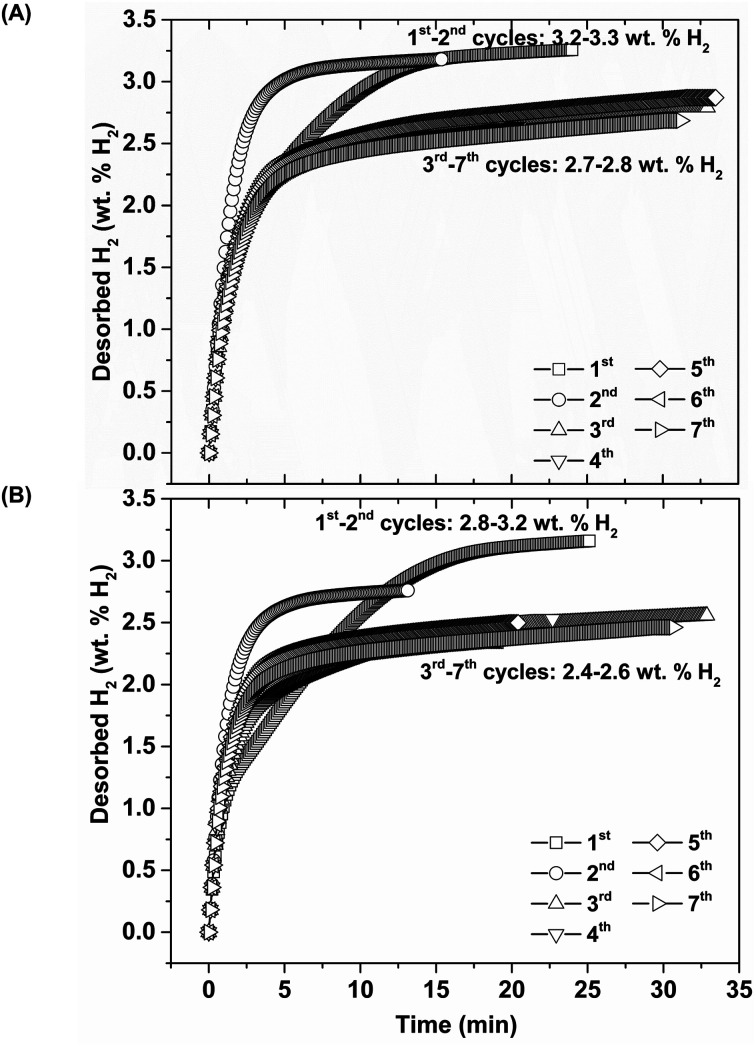
Dehydrogenation kinetics and reversible capacities upon 7 hydrogen release and uptake cycles of S1 (A) and S2 (B).

Thus, lower *P*_sys_ (1.13 and 0.4 bar H_2_ for S1 and S2, respectively) than *P*_eq_ at these *T*_eq_ encourages dehydrogenation of both samples. Afterwards rehydrogenation is carried out at isothermal condition (*T*_set_ = 315 °C) under 16 bar H_2_. By applying hydrogen pressure, *T*_sample_ of both S1 and S2 enhance rapidly to *T*_eq_ = 332 and 351 °C, respectively, due to fast exothermic reaction (Fig. 3). Rehydrogenations of both samples complete within 11 min, assured by the reduction of *T*_sample_ to the initial temperature. Under comparable *P*_sys_ (16 bar H_2_), different *T*_eq_ values detected during hydrogenation of S1 and S2 suggest the alteration of reversible phases and reaction pathways. In the case of the 2^nd^ dehydrogenation, S1 and S2 reveal fast temperature reduction to comparable *T*_eq_, *P*_sys_, and reaction time of 308–311 °C, 0.8–1.0 bar H_2_, and 10–11 min, respectively (Fig. 3). Afterwards, dehydrogenation kinetics, capacities, and reversibility upon 7 de/rehydrogenation cycles of S1 and S2 are investigated. During the 1^st^ dehydrogenation, hydrogen capacities of S1 and S2 are comparable of 3.2–3.3 wt% H_2_, but S1 shows faster dehydrogenation rate than S2 (Fig. 4). Considering the 2^nd^ dehydrogenation, kinetic properties of both samples are improved with respect to the 1^st^ cycle. Reversible capacity in the 2^nd^ cycle of S1 is maintained as 3.3 wt% H_2_, while that of S2 reduces to 2.8 wt% H_2_ (Fig. 4). Upon the 3^rd^–7^th^ cycles, kinetic properties of both samples are stable, but their storage capacities reduce to 2.7–2.8 and 2.4–2.6 wt% H_2_ for S1 and S2, respectively.

**Fig. 5:**
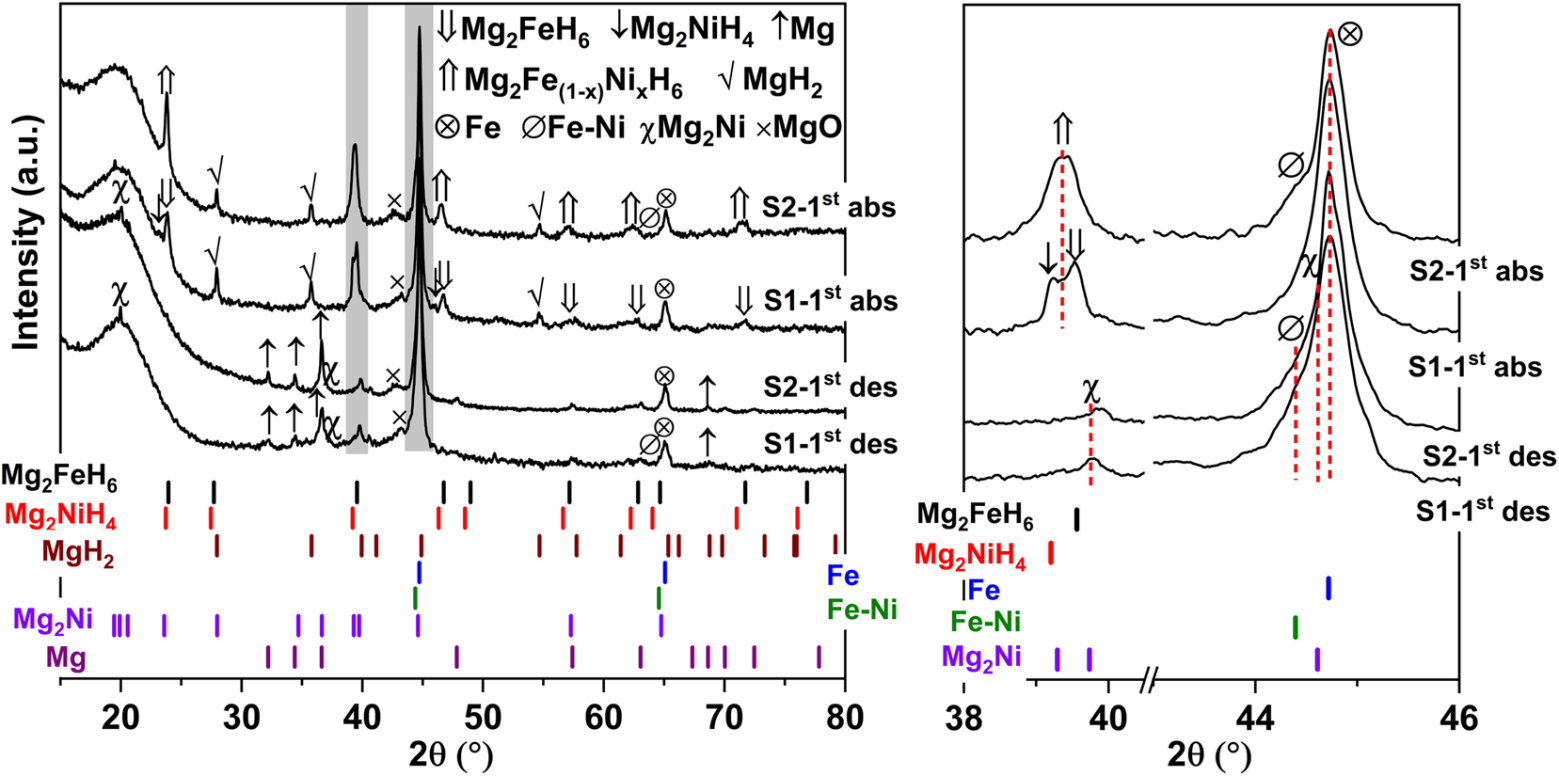
PXRD spectra of the 1^st^ de/rehydrogenated S1 and S2.

Furthermore, phase compositions of S1 and S2 after the 1^st^ de/rehydrogenation are investigated by PXRD technique. From Fig. 5, the 1^st^ dehydrogenated S1 and S2 reveal comparable diffractions of Mg, Mg_2_Ni, Fe, MgO, and Fe–Ni alloy. Considering phase compositions of as-prepared and the 1^st^ dehydrogenated samples of S1 and S2, Mg and Fe are obtained from the dehydrogenation of MgH_2_ and Mg_2_FeH_6_ (eqn (6) and (7)), while Mg_2_Ni is from the decomposition of Mg_2_NiH_4_ (reverse reaction of eqn (5)).

6 MgH_2(S)_ → Mg_(S)_ + H_2(g)_

7 Mg_2_FeH_6(s)_ → 2Mg_(s)_ + Fe_(s)_ + 3H_2(g)_

For the 1^st^ rehydrogenation, the formations of MgH_2_, Mg_2_NiH_4_, Mg_2_FeH_6_, and MgO are observed in S1. In the case of S2, the 1^st^ rehydrogenated sample reveals the diffractions of MgH_2_, Fe, Fe–Ni alloy, and MgO as well as Mg_2_Fe_(1−*x*)_Ni_*x*_H_6_, shown as a new diffraction peak locating between those of Mg_2_FeH_6_ and Mg_2_NiH_4_.^23,25^ The formations of MgH_2_, Mg_2_FeH_6_, and Mg_2_NiH_4_ in S1 confirm rehydrogenation of Mg, MgH_2_ + Fe, and Mg_2_Ni, respectively (reverse reactions of eqn (6) and (7) as well as eqn (5)). Besides, it was reported that Mg_2_NiH_4_ was able to be synthesized by hydrogenating the mixture of coarse-grained Mg and Ni(Fe) nanoparticles and most of Ni(Fe) transformed to α-Fe when the reaction completed (eqn (8)).^33^ Thus, the reduction of Fe–Ni alloy together with the increment of Fe after the 1^st^ rehydrogenation of S1 (Fig. 5) can be explained by the reaction between Fe–Ni alloy and MgH_2_ to form Mg_2_NiH_4_. In the case of the 1^st^ rehydrogenated S2, hydrogenations of Mg into MgH_2_ (reverse reaction of eqn (6)) and Mg_2_Ni + Mg_2_FeH_6_ into Mg_2_Fe_(1−*x*)_Ni_*x*_H_6_ (eqn (9))^23,25^ are observed. Significantly enhanced diffraction of Fe–Ni alloy and irreversibility of Mg_2_NiH_4_ upon the 1^st^ hydrogenation of S2 suggest the increase of solid solution of Fe and Ni as well as no reaction between MgH_2_ and Fe–Ni alloy (eqn (8)). Reaction pathways upon the 1^st^ de/rehydrogenation are summarized in Table 1.

8 2MgH_2(s)_ + Ni(Fe)_(s)_ → Mg_2_NiH_4(s)_ + α-Fe_(s)_

9 *x*Mg_2_Ni_(s)_ + (1 − *x*)Mg_2_FeH_6(s)_ + 3*x*H_2(g)_ → Mg_2_Fe_(1−*x*)_Ni_*x*_H_6(s)_

**Table 1:** Reaction pathways and phase compositions of S1 and S2 during the 1^st^ de/rehydrogenation

Samples	Possible reaction pathways and phase compositions
**S1**	
As-prepared	MgH_2_ + Mg_2_FeH_6_ + Mg_2_NiH_4_
1^st^ desorbed	Mg_2_FeH_6_ → 2MgH_2_ + Fe + H_2_
	MgH_2_ → Mg + H_2_
	Mg_2_NiH_4_ → Mg_2_Ni + 2H_2_
	Fe + Ni → Fe–Ni
1^st^ absorbed	Mg + H_2_ → MgH_2_
	2MgH_2_ + Fe + H_2_ → Mg_2_FeH_6_
	Mg_2_Ni + 2H_2_ → Mg_2_NiH_4_
	2MgH_2_ + Fe–Ni → Mg_2_NiH_4_ + Fe^33^

**S2**	
As-prepared	Mg_2_FeH_6_ + Mg_2_NiH_4_ + Fe–Ni
1^st^ desorbed	Mg_2_FeH_6_ → 2MgH_2_ + Fe + H_2_
	MgH_2_ → Mg + H_2_
	Mg_2_NiH_4_ → Mg_2_Ni + 2H_2_
	Fe–Ni (comparable to as-prepared state)
1^st^ absorbed	Mg + H_2_ → MgH_2_
	2MgH_2_ + Fe + H_2_ → Mg_2_FeH_6_·*x*Mg_2_Ni + (1 − *x*)Mg_2_FeH_6_ + 3*x*H_2_ → Mg_2_Fe_(1−*x*)_Ni_*x*_H_6_
	Fe + Ni → Fe–Ni

**Fig. 6:**
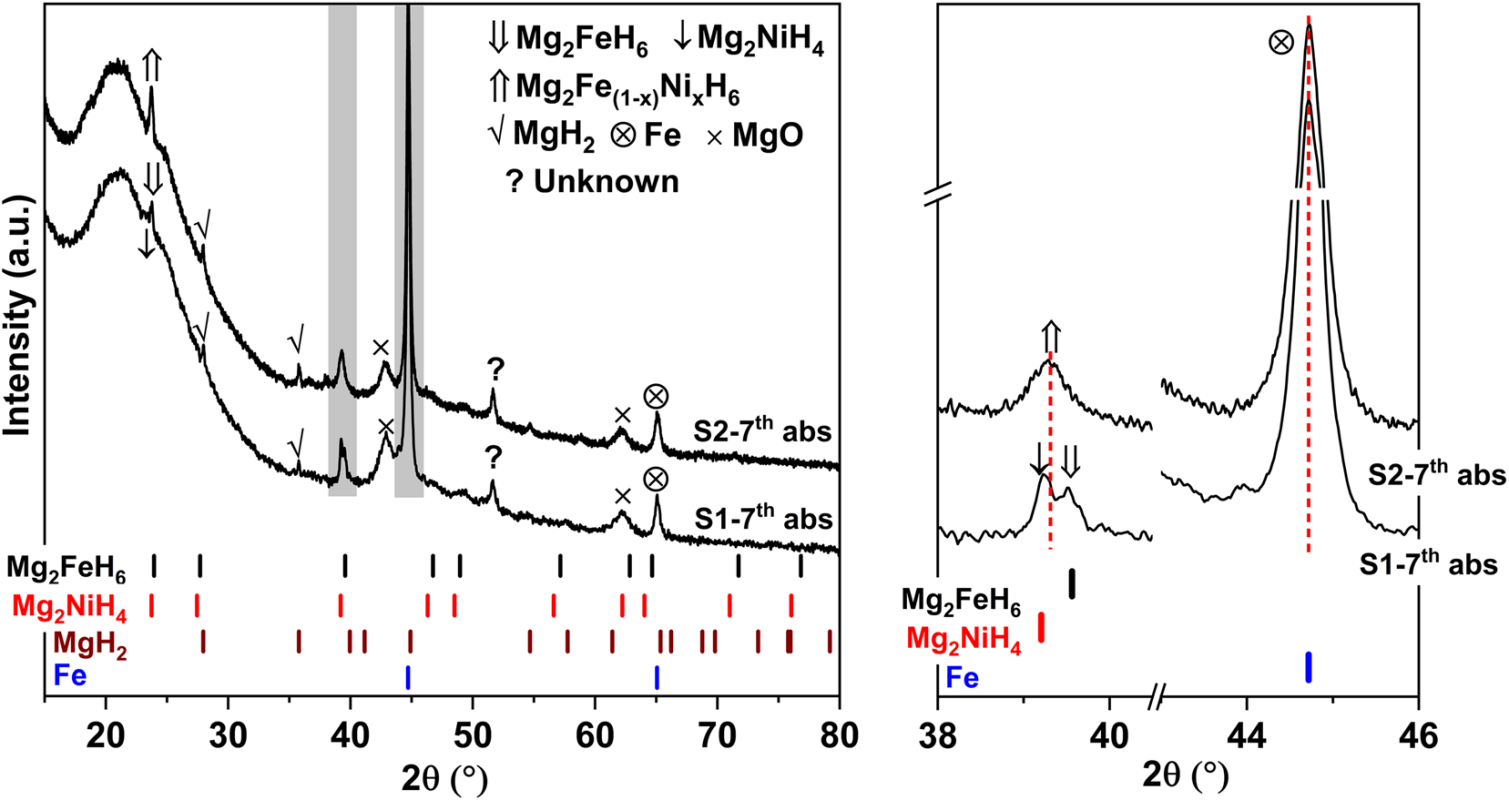
PXRD spectra of the 7^th^ rehydrogenated S1 and S2.

Due to the changes of reaction pathways and phases formed during the 1^st^ de/rehydrogenation of S1 and S2 (Fig. 5 and Table 1), temperature profiles during the 1^st^ endothermic desorption and exothermic absorption of S1 and S2 are different (Fig. 3). Effective reproducibility of several hydrides in S1 (MgH_2_ + Mg_2_FeH_6_ + Mg_2_NiH_4_) probably maintains reversible hydrogen capacities upon 2 cycles (∼3.3 wt% H_2_) (Fig. 4A). Moreover, phase compositions of the 7^th^ rehydrogenated samples of S1 and S2 are characterized by PXRD technique to describe the reduction of hydrogen capacities upon cycling (Fig. 4). From Fig. 6, both rehydrogenated samples show comparable diffractions of MgH_2_, Fe, MgO, and unknown phase. Meanwhile, each sample shows different phases of Mg_2_FeH_6_ + Mg_2_NiH_4_ and Mg_2_Fe_(1−*x*)_Ni_*x*_H_6_ for the 7^th^ rehydrogenated S1 and S2, respectively. Upon cycling, significant amount of unreacted Fe with respect to the reversible hydrides is observed from both samples. The latter explains the deficient hydrogen capacities of both samples upon the 3^rd^–7^th^ cycles (Fig. 4).

**Fig. 7:**
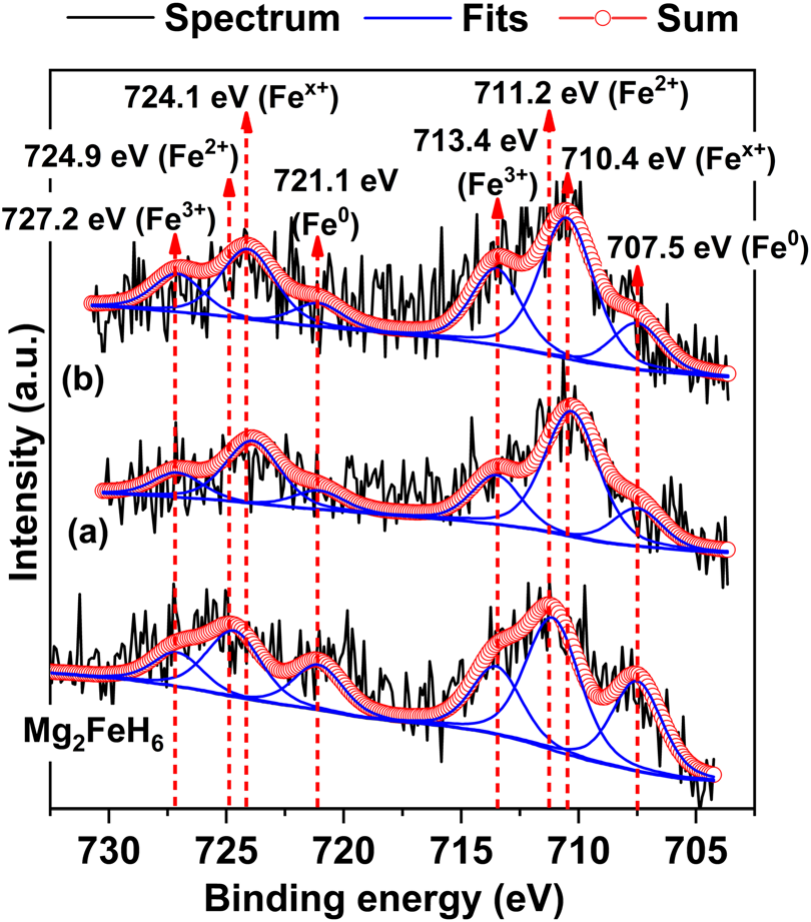
Fe 2p XPS spectra of as-prepared Mg_2_FeH_6_ as well as the 1^st^ (a) and the 7^th^ (b) rehydrogenated S2.

To confirm the formation of Mg_2_Fe_(1−*x*)_Ni_*x*_H_6_ in the 1^st^ and 7^th^ rehydrogenated S2, Fe 2p XPS experiments are carried out. From Fig. 7, Fe 2p XPS spectrum of as-prepared Mg_2_FeH_6_ shows the characteristic peaks of Fe^0^ (707.5 and 721.1 eV), Fe^2+^ (711.2 and 724.9 eV), and Fe^3+^ (713.4 and 727.2 eV), belonging to metallic Fe, Mg_2_FeH_6_, and Fe_2_O_3_, respectively.^34,35^ The signal of metallic Fe is attributed to unreacted Fe during Mg_2_FeH_6_ preparation, while that of Fe_2_O_3_ is likely due to the oxidation of Fe during the measurements. For the 1^st^ and 7^th^ rehydrogenated S2, Fe 2p XPS peaks of Fe^0^ and Fe^3+^ of metallic Fe and Fe_2_O_3_, respectively, are observed at comparable binding energies with as-prepared Mg_2_FeH_6_. Besides, the new peaks of Fe^*x*+^ (710.4 and 724.1 eV) locating at lower binding energies than Fe^2+^ are detected (Fig. 7(a) and (b)). This suggests the formation of another Fe-containing phase with lower oxidation state than 2+. Because the energy resolution of XPS measurements is 0.5 eV, the binding energy difference between Fe^2+^ and Fe^*x*+^ (∼0.8 eV) is sufficient to imply that the energy shift is due to phase changes. Once partial substitution of Ni for Fe in Mg_2_FeH_6_ to form Mg_2_Fe_(1−*x*)_Ni_*x*_H_6_ occurs, the oxidation state of Fe reduces from Fe^2+^ to Fe^*x*+^ (0 < *x* < 2). Thus, the appearance of Fe^*x*+^ likely confirms the formation of Mg_2_Fe_(1−*x*)_Ni_*x*_H_6_ in the 1^st^ and 7^th^ rehydrogenated S2.

**Fig. 8:**
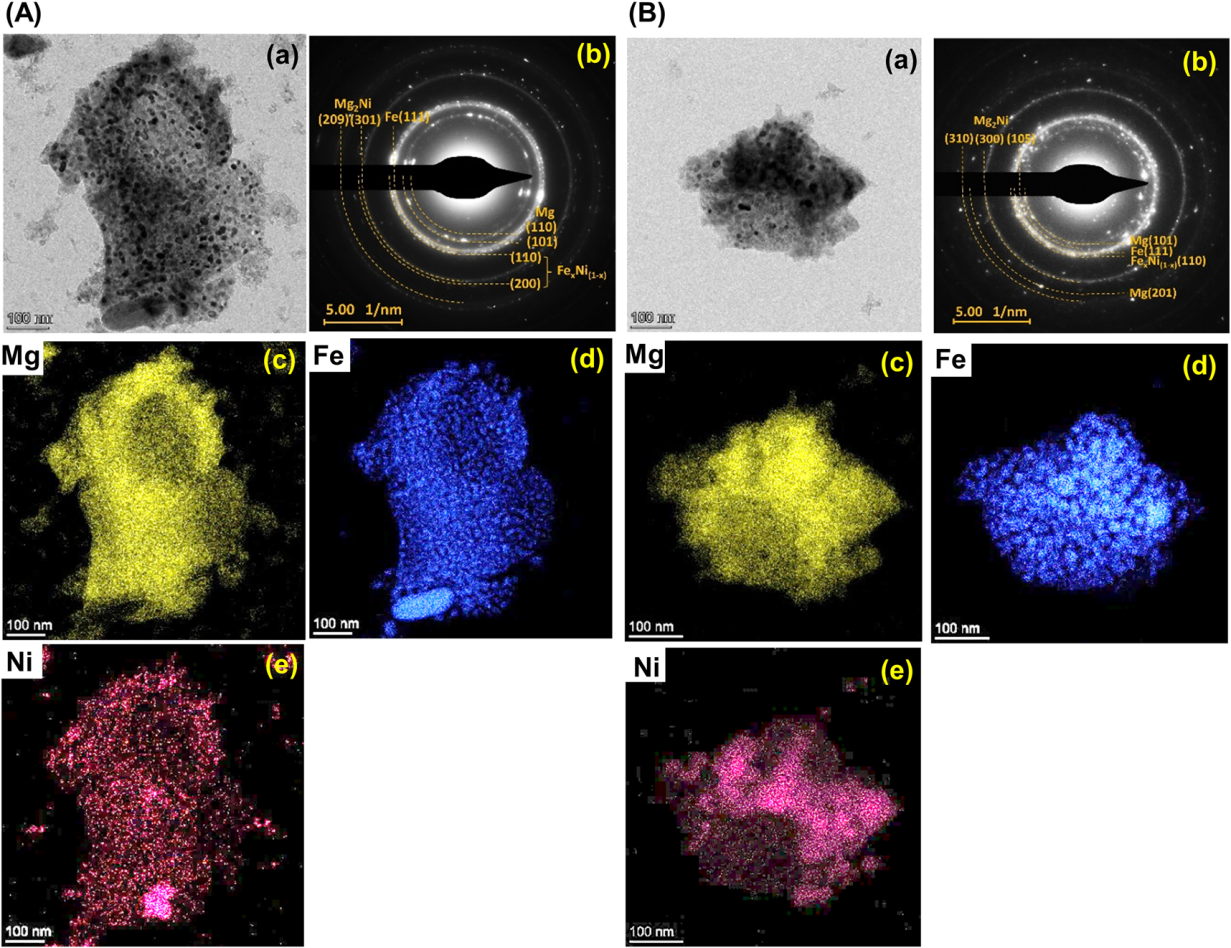
The bright field TEM micrographs (a), the corresponding selected area electron diffraction (SAED) pattern (b), and elemental mapping of Mg (c), Fe (d), and Ni (e) of the 1^st^ dehydrogenated samples of S1 (A) and S2 (B).

Furthermore, it should be mentioned that phase compositions in the 1^st^ dehydrogenated samples of S1 and S2 are comparable (*i.e.*, Mg, Mg_2_Ni, Fe–Ni alloy, and Fe) (Fig. 5). However, the reaction pathways during the 1^st^ rehydrogenation of these samples are different, affecting reversible hydrogen capacities (Table 1 and Fig. 4). This might relate to contacts and distribution of the reactive phases in the bulk samples. Therefore, microstructural analyses of the 1^st^ dehydrogenated S1 and S2 are investigated by TEM, electron diffraction, and EDS mapping. TEM image of the 1^st^ dehydrogenated S1 shows that at least two different phases are well distributed in the nanometer scale (Fig. 8A(a)). The corresponding SAED pattern confirms the presence of Mg, Mg_2_Ni, and Fe–Ni (Fig. 8A(b)), in accordance with PXRD result (Fig. 5). EDS maps reveal excellent distribution of Mg, Fe, and Ni in the sample bulk (Fig. 8A(c) and (e)). These results suggest good contacts among Mg, Fe, Mg_2_Ni, and Fe–Ni in the 1^st^ dehydrogenated S1. This likely promotes the formation of Mg_2_FeH_6_ and Mg_2_NiH_4_ upon rehydrogenation (Fig. 5 and Table 1). In the case of the 1^st^ dehydrogenated S2, TEM micrograph shows significant particle agglomeration (Fig. 8B(a)) with comparable phase compositions to S1 (SAED result in Fig. 8B(b)). From EDS maps, Mg and Ni occupying comparable location show well-distributed nanoparticles with partially dense agglomeration (Fig. 8B(c) and (e)), while Fe shows good distribution of sintered particles (Fig. 8B(d)). These distributions either of nanoparticles or sintered particles found in Mg, Ni, and Fe maps lead to the homogeneous reversibility of MgH_2_, Mg_2_FeH_6_, and Fe–Ni alloy all over the sample bulk. The positions with Mg and Ni agglomeration, probably containing high density of Mg_2_Ni benefit for hydrogenation of Mg_2_FeH_6_ + Mg_2_Ni to form Mg_2_Fe_(1−*x*)_Ni_*x*_H_6_ (eqn (9)). Thus, using different Ni sources (metallic Ni or Mg_2_NiH_4_) as staring material affects the contacts among active phases. S1 using metallic Ni shows better distribution of metal nanoparticles than S2, which Ni is from Mg_2_NiH_4_. The 1^st^ dehydrogenated S1 with good metal distribution reproduces individual hydrides (Mg_2_FeH_6_ and Mg_2_NiH_4_) upon rehydrogenation. For the 1^st^ dehydrogenated S2, agglomeration of Mg_2_Ni (from direct decomposition of Mg_2_NiH_4_), which is in good contacts with Mg and Fe favors the formation of Mg_2_Fe_(1−*x*)_Ni_*x*_H_6_. Therefore, the distribution and contacts among metal nanoparticles results in different reaction pathways upon de/rehydrogenation and reversible hydrogen capacities.

## 4. Conclusions

The effects of Ni precursors (metallic Ni or Mg_2_NiH_4_) on the formation of Mg–Fe–Ni hydrides were studied and the de/rehydrogenation kinetics and reversibility of the obtained samples were investigated. The mixtures of MgH_2_ + Mg_2_FeH_6_ + Mg_2_NiH_4_ and Mg_2_FeH_6_ + Mg_2_NiH_4_ were obtained from the as-prepared samples with metallic Ni and Mg_2_NiH_4_, respectively. Although both samples released comparable hydrogen during the 1^st^ cycle (3.2–3.3 wt% H_2_), the reduction of dehydrogenation temperature (Δ*T* = 12 °C) and faster kinetics were obtained from the as-prepared sample with metallic Ni. After the 1^st^ dehydrogenation, similar phase compositions of Mg, Mg_2_Ni, Fe, and Fe–Ni alloy were found in both samples. Nevertheless, different Ni precursors altered phase compositions and reaction pathways during rehydrogenation. The reversible phases of the sample with metallic Ni were MgH_2_, Mg_2_FeH_6_, and Mg_2_NiH_4_, while those of the sample with Mg_2_NiH_4_ were MgH_2_ and Mg_2_Fe_(1−*x*)_Ni_*x*_H_6_. These recovered phases affected reversible capacities. For example, hydrogen capacities during the 2^nd^–7^th^ cycles of the sample with metallic Ni were 2.7–3.2 wt% H_2_, while those of the sample with Mg_2_NiH_4_ reduced to 2.4–2.8 wt% H_2_. Deficient reversible capacities of both samples, especially after the 3^rd^ cycles could be described by significant amount of unreacted Fe. Considering microstructural analyses, the sample with metallic Ni contained well-distributed nanoparticles of all metals, benefiting for individual reversibility of MgH_2_, Mg_2_FeH_6_ and Mg_2_NiH_4_. For the sample with Mg_2_NiH_4_, partial agglomeration of Mg and Ni at comparable location, likely belonging to Mg_2_Ni favoured the formation of Mg_2_Fe_(1−*x*)_Ni_*x*_H_6_. Due to the recovery of multiple hydride phases, hydrogen capacities upon cycling of the sample with metallic Ni was significant.

## Author contributions

Palmarin Dansirima: sample preparation, characterizations, sharing idea, data analysis, and manuscript writing. Sophida Thiangviriya: sample preparation and characterizations. Praphatsorn Plerdsranoy: characterizations. Narong Chanlek: XPS measurements and analysis. Rapee Utke: conceptualization, supervision, data analysis, manuscript writing, and reviewing and editing.

## Conflicts of interest

There are no conflicts to declare.

## Supplementary Material
