# Correction: Effects of Ni precursors on the formation of Mg–Fe–Ni intermetallic hydrides, kinetics, and reversibility

**DOI:** 10.1039/d3ra90070c

**Published:** 2023-08-09

**Authors:** Palmarin Dansirima, Sophida Thiangviriya, Praphatsorn Plerdsranoy, Narong Chanlek, Rapee Utke

**Affiliations:** a School of Chemistry, Institute of Science, Suranaree University of Technology Nakhon Ratchasima 30000 Thailand rapee.g@sut.ac.th; b Synchrotron Light Research Institute (Public Organization) Nakhon Ratchasima 30000 Thailand

## Abstract

Correction for ‘Effects of Ni precursors on the formation of Mg–Fe–Ni intermetallic hydrides, kinetics, and reversibility’ by Palmarin Dansirima *et al.*, *RSC Adv.*, 2023, **13**, 16926–16934, https://doi.org/10.1039/D3RA01914D.

The authors regret that there was an error in Table 1. The correct version of Table 1 is presented below.

**Table 1:** Reaction pathways and phase compositions of S1 and S2 during the 1st de/rehydrogenation

Samples	Possible reaction pathways and phase compositions
**S1**	
As-prepared	MgH_2_ + Mg_2_FeH_6_ + Mg_2_NiH_4_
1st desorbed	Mg_2_FeH_6_ → 2MgH_2_ + Fe + H_2_
	MgH_2_ → Mg + H_2_
	Mg_2_NiH_4_ → Mg_2_Ni + 2H_2_
	Fe + Ni → Fe–Ni
1st absorbed	Mg + H_2_ → MgH_2_
	2MgH_2_ + Fe + H_2_ → Mg_2_FeH_6_
	Mg_2_Ni + 2H_2_ → Mg_2_NiH_4_
	2MgH_2_ + Fe–Ni → Mg_2_NiH_4_ + Fe^33^

**S2**	
As-prepared	Mg_2_FeH_6_ + Mg_2_NiH_4_ + Fe–Ni
1st desorbed	Mg_2_FeH_6_ → 2MgH_2_ + Fe + H_2_
	MgH_2_ → Mg + H_2_
	Mg_2_NiH_4_ → Mg_2_Ni + 2H_2_
	Fe–Ni (comparable to as-prepared state)
1st absorbed	Mg + H_2_ → MgH_2_
	2MgH_2_ + Fe + H_2_ → Mg_2_FeH_6_
	*x*Mg_2_Ni + (1 − *x*)Mg_2_FeH_6_ + 3*x*H_2_ → Mg_2_Fe_(1−*x*)_Ni_*x*_H_6_
	Fe + Ni → Fe–Ni

The Royal Society of Chemistry apologises for these errors and any consequent inconvenience to authors and readers.

## Supplementary Material

